# Palm springs on the Rio Grande: Insight into Archaic forager plant use from phytoliths recovered from a Late Holocene alluvial section in northern New Mexico

**DOI:** 10.1371/journal.pone.0258231

**Published:** 2021-10-12

**Authors:** Alison Damick, Arlene Rosen, Scott Ortman

**Affiliations:** 1 Department of Anthropology, University of Texas at Austin, Austin, Texas, United States of America; 2 Department of Anthropology, University of Colorado at Boulder, Boulder, Colorado, United States of America; University at Buffalo - The State University of New York, UNITED STATES

## Abstract

In this study we present new evidence from phytolith studies for the presence of *Sabal sp*. (likely *minor*), an allochthonous plant, around Tesuque Creek in northern New Mexico during the early part of the Late Holocene, in the vicinity of known Late Archaic hunter-gatherer communities using the area at that time. We analyzed phytoliths from sediments taken from an alluvial section on the east side of Tesuque Creek dating to c. 3600–2400 cal. BP. The phytoliths demonstrated a change over time from a succulent dominated landscape to a shrubby one, with the later introduction of high densities of palmetto phytoliths associated with marshy deposits and adjacent burn levels. This evidence suggests a more diverse resource landscape available to local hunter-gatherer groups than previously understood, and may have implications for the early management of microenvironments, plant communities. This evidence demonstrates the value of phytolith analysis from alluvial sections for understanding human land and plant use practices over time. Our study provides a new perspective on what resources and land use areas were available for Archaic peoples inhabiting the area, and how they may have experimented with managing lesser known types of wild plant resources before the establishment of the triad of crops from Mesoamerica. This opens up new avenues for understanding the landscapes, land use practices, and environmental impacts of pre-agricultural communities in the northern Rio Grande and in other semi-arid environments worldwide.

## Introduction

Recent research has shown that full-time foragers in numerous locations around the world cultivated wild plants for at least 10,000 years before the emergence of truly domesticated cultigens and the adoption of farming [1–4]. In most cases, research on this phenomenon has focused on wild progenitors of domesticated cultigens known as “Founder Crops” such as wheat in Southwest Asia, rice in China, and maize in the Americas [5–12]. These crops attract research attention because they are among the most important modern cultigens, but for foragers, other types of plants were of equal importance to their subsistence regime. Ethnohistoric research has shown that foragers also tended and cultivated a range of plants that were not subsequently domesticated [13–15]. Here we provide compelling paleoecological evidence for the availability of an important species of wild plant in northern New Mexico, which is outside its endemic range, and therefore may suggest Archaic forager participation in maintaining its growth in the region.

Direct archaeological evidence for cultivation of non-domesticates by past foragers is rare [16–22]. Two main factors contribute to the scarcity of evidence that can pinpoint pre-agricultural cultivation of wild plants. First, many archaeobotanical studies derive from assemblages collected at archaeological sites, where wild plants could represent collection rather than cultivation. Second, evidence for local wild plants found in paleoecological samples, for instance from pollen cores, could indicate vegetation changes due to the effects of climatic fluctuations and natural environmental change rather than human activity. One solution to these problems is the identification of an introduced non-local wild plant growing in a landscape context exploited by forager populations, which would clearly point to anthropogenic manipulation of that species. For this study, we examined the geomorphological and microbotanical (phytolith) record of a Late Holocene alluvial section in Tesuque Creek, an area adjacent to, but outside of known forager archaeological sites. From this work, we report the first direct evidence for the presence of dwarf palmetto (*Sabal minor*), a non-native plant, in the northern Rio Grande area of New Mexico, USA, in an area accessed by Late Archaic foragers.

The detailed phytolith record obtained allowed us to reconstruct the local landscape, environmental history, and changing plant communities from the period of Late Archaic forager occupation through the beginning of cultivation. Our results add to emerging evidence that low-level tending and cultivation of wild plants was widespread in foraging societies and extends to taxa that were not subsequently domesticated. It also suggests possible early cultivation of wild species in regions beyond which they are endemic. This serves as a baseline for understanding the dynamics of plant resource manipulation by preagricultural foragers living in this region as a prelude to understanding how and why agriculture was ultimately adopted by more recent inhabitants of the region.

## Study area and resources

Throughout the Late Holocene, the vegetation in the northern Rio Grande Basin of New Mexico has been dominated by piñon and juniper, with a component of Agavoideae in the form of yucca. There are also steppic grasses such as *Stipa* along with shrubs such as chenopods and amaranth [19, 23, 24]. These plants provided a wide range of resources for both foragers and agriculturalists, fulfilling both nutritional and material needs. Vierra and Ford have suggested that such plants were sometimes cultivated by Archaic foragers around cave sites in particular [12, 19, 25, 26].

**Fig 1:**
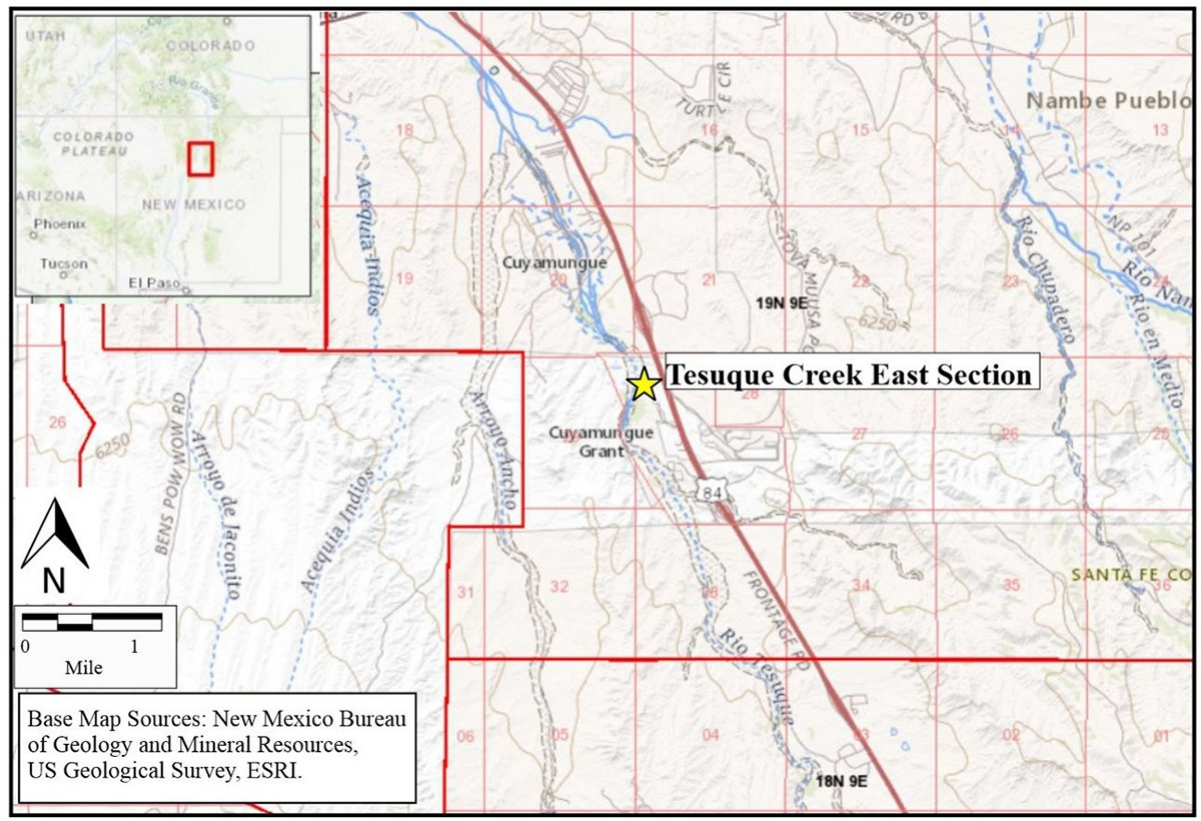
Map of the location of the Tesuque Creek East Section.

In order to track key elements of vegetation history, and the impact of human occupation in this region, we focused our preliminary work on a small catchment of the Tesuque Creek alluvial system in northern New Mexico (Fig 1). The study site is located on the Pojoaque Pueblo land grant, in the southernmost part of the Española Basin, along the lower catchment area of Tesuque Creek as it passes Pojoaque Pueblo and the ancestral Tewa village of Cuyumungué. Today, precipitation in the study area averages around 350 mm per annum, with the heaviest rainfall occurring during the summer months.

The section that we studied is located upstream from a known Late Archaic archaeological site [27, 28]. We collected thirty sediment samples from this five-meter high alluvial section and analyzed them for phytolith remains. These samples, deposited from about 3700–2600 cal BP, provided new information about human impact on vegetation patterns within this alluvial floodplain. The microbotanical analyses produced large numbers of phytoliths dominated by Agavoideae in the lower portion of the section. Shrubs and grasses increased in the upper part of the section. There was a strong presence of palm phytoliths likely deriving from the dwarf palmetto, *Sabal minor*, in the upper units associated with seasonally marshy floodplain deposits and associated burn levels. Most of these plants are native to this region and are currently present along the modern floodplains. While the palmettos are not endemic to northern New Mexico, they grow in moist micro-environments as far north as Oklahoma and Texas and as far east as Florida up to South Carolina. The *Sabal* palmetto could have provided an important resource for Late Archaic inhabitants of New Mexico, yielding an edible starchy stalk, roots used for soap-making, and fronds and fibers used for a wide variety of useful material purposes. We suggest that these palmettos were likely cultivated and maintained by the Archaic inhabitants of this region, and may indeed have been opportunistically planted by them.

### Late Holocene paleoclimate

The paleoclimate was a significant driver of stream flow, water resources and vegetation composition and coverage. Recent climate literature indicates that in the mid-Holocene altithermal, the climate was warm and dry. Climate proxies indicate a shift to generally wetter, cooler conditions in the Late Holocene [29–32]. This cooler/moister period begins around 3,000 cal BP in the Sangre de Cristo Mountains [33, 34], although Hall’s 2017 regional summary dates this “Late Holocene Wet Period” from 4500–1000 cal BP. This would have led to greater runoff into the valley alluvial systems, and higher water tables. This period was characterized as a neo-glaciation event at higher elevations in the mountains and coincides with the expansion of piñon pine forests and the retraction of grassland ranges [33, 35–37]. These climatic conditions would seem to suggest an environment that was too cold to sustain a palmetto population. However, *Sabal minor* is the hardiest and most frost-tolerant of all the palmettos [38].

### Overview of Late Archaic occupation and plant use

In northern New Mexico and most of the Southwest, the Late Archaic spans c. 3500–1500 BP, post-dating the Middle Archaic (c. 5500–3500 BP) and the Early Archaic (c. 8500–5500 BP) [36, 39]. The Archaic Period landscape was populated by nomadic and semi-nomadic foragers who exploited a broad range of plants and animals. The Late Archaic marks the emergence of new technologies and subsistence patterns demonstrating complex land management strategies that are considered foundational in the development of agricultural societies throughout the region [36, 40–42]. Vierra and Ford have suggested low level cultivation of wild plants and imported cultigens around cave sites [19].

In the northern Rio Grande, Archaic plant use patterns generally reflect regional trends towards broad-spectrum foraging, which becomes more specialized over time as populations begin low-level management of their landscapes and resources. The cooler and wetter late Holocene environmental conditions presented opportunities for Archaic Period foragers who inhabited this region. By the Late Archaic, the archaeobotanical records provide some evidence of the introduction of early cultigens, although these arrive late to northern and eastern New Mexico and remain minimal contributors to subsistence for quite a long time relative to other parts of the region.

Cheno-ams and wild weedy plants appear in archaeobotanical records alongside piñon as important Archaic resources providing starch and protein to local foragers [43]. Succulents, particularly *Yucca baccata* and possibly other Agavoideae, also provided essential calories and starch, and seem to have served both subsistence and technical purposes throughout the Southwest [44–48]. Eventually starch-rich Mesoamerican cultigens such as maize, cucurbits (squash and gourd), and *Phaseolus* spp. (various beans) were incorporated into the local subsistence system, but remained less important resources for centuries [19, 22, 49]. Indeed, the rarity of these early introduced cultigens until quite late in the Archaic, and their slow incorporation into existing subsistence systems, is a distinctive and notable feature of the archaeobotanical record for Eastern and North-Eastern New Mexico [12, 39, 50, 51]. The archaeobotanical record also indicates a persistent focus on resources from alluvial catchment zones throughout the Archaic [52–54]. This emphasizes the importance of studying these alluvial zones to understand the diversity of plant communities within these microenvironments and how they responded to human plant exploitation and management.

## Results

**Fig 2:**
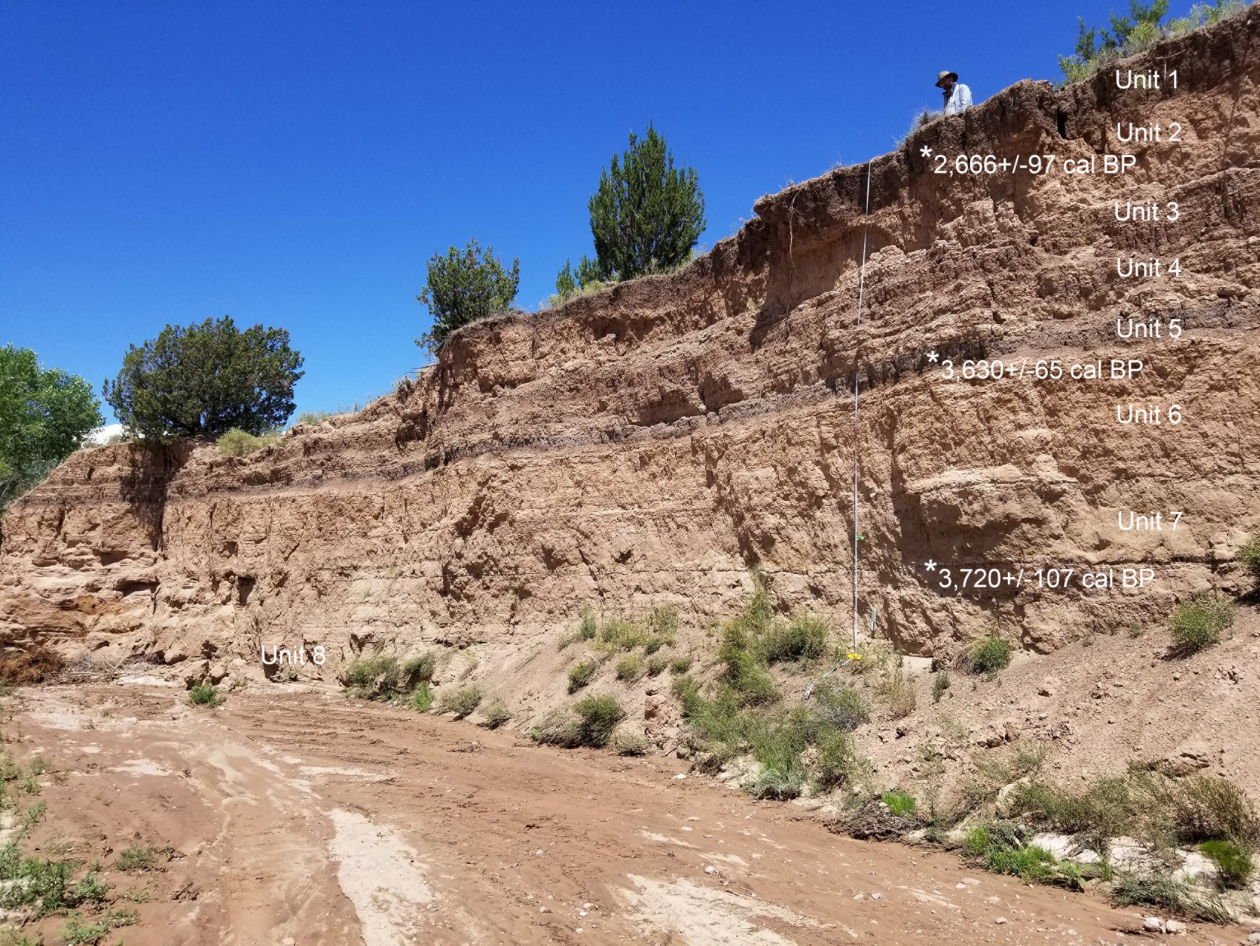
Tesuque Creek East Section showing units and location of C^14^ dates.

The modern channel of Tesuque Creek generally supports an ephemeral stream with some limited perennial flow throughout most of the year. Today, this portion of the arroyo is deeply incised with strong flash flooding after storms, but the flow can no longer reach bankfull conditions or develop a floodplain in our study area. We identified exposures of alluvial deposits that were five- eleven meters deep on the east and west faces of the drainage. One of the geological sections of this study, Tesuque Creek East (TCE) (N35, 51’ 29.2”; W106, 0.0’, 02.9”; elev. 1860 masl), provided an approximately five-meter deep sequence of Late Holocene sediment deposition. The lowermost portion of the section consisted primarily of sandy silts with some laminated bedding, indicating deposits from an aggrading floodplain of a primarily perennial stream, with some overbank levee deposition. In the upper three meters of the section there are sets of organic-rich clayey silts indicating ponding and seasonal marsh deposits on these ancient floodplains. The sediment units were described, and sampled for ^14^C dates, sediment analyses and phytolith determinations. We identified eight sediment units which reflect changes in the depositional regime, including marshy deposits (Units 1, 3, and 5), near-channel overbank sediments (Units 2, 4, 6, and 8), and one unit with sets of both types of depositional microenvironments (Unit 7). Small well-sorted and rounded channel gravels appear at intervals throughout the section indicating periods of well-sustained perennial flow (Fig 2). Throughout the section there are lenses of ash and charcoal indicating burned surfaces. These are commonly associated with the marshy facies of these deposits (Units 1, 3, 5, and 7), although one burn level appears in Unit 6. For the purposes of this paper we primarily will discuss the results of the phytolith analysis (See S1 File for full counts).

### Phytolith analysis

We collected 30 sediment samples for phytolith analysis from every unit throughout the TCE Geo-Section. The full results of all phytolith forms counted are provided in the S1 File as absolute numbers of phytoliths per gram of sediment. Generally, we found substantial quantities of phytoliths in most samples. Most of the phytoliths were silica bodies from monocotyledons (grasses, sedges, palms), with the exception of the shapeless silica masses termed “siliceous aggregates” and “platelets” which form within the woody parts of dicotyledonous trees and shrubs [55]. We also found large quantities of raphids from succulents in a number of the samples (S1 File).

Here we wish to highlight four categories of plant types that are indicative of vegetation and landscape shifts through time, and provide information about the types of plants which might have been available for exploitation by the native inhabitants of the region during the Late Archaic Period. These four categories include 1) Gramineae 2) General dicotyledonous shrubs, 3) Agavaceae (most likely yucca), and 4) Palmettos within the more mesic sediment units.

**Fig 3:**
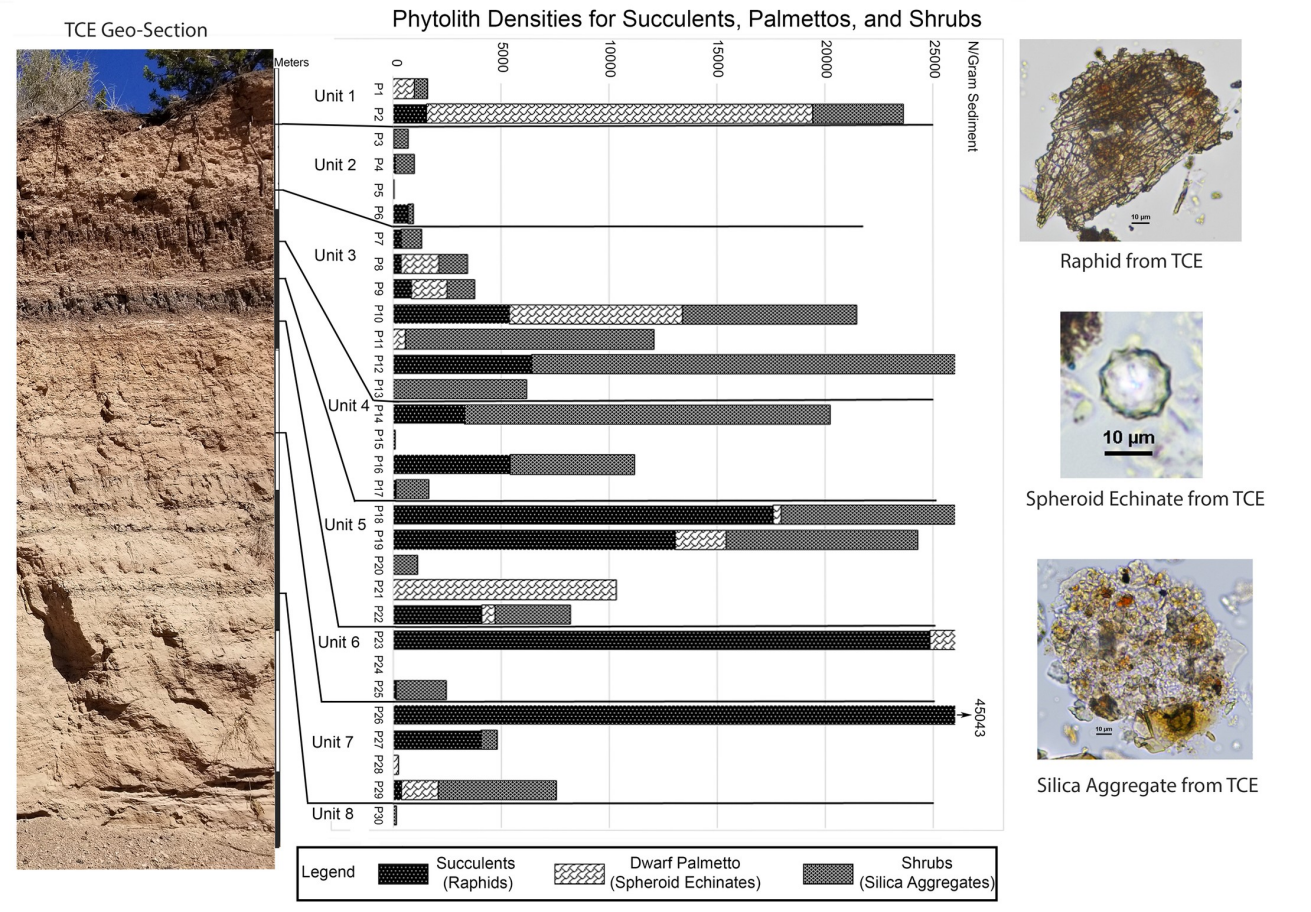
Tesuque Creek East Section showing with phytolith density results (n/gram). Phytolith densities are shown for proxies for shrubs, palmetto, and agave, as well as photos of these representative morphotypes.

**Fig 4:**
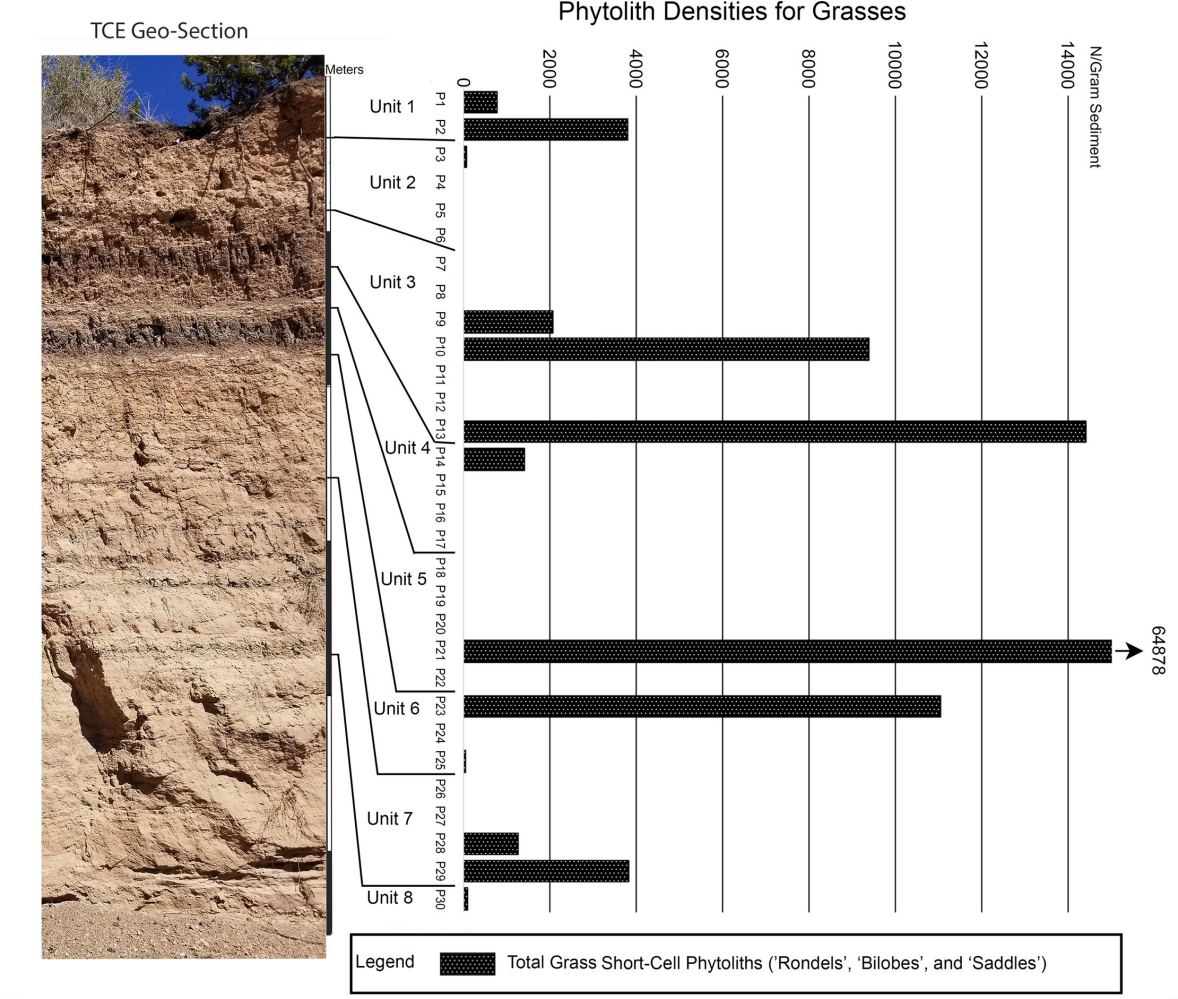
Tesuque Creek East Section with phytolith density results (n/gram) for the grasses.

These plant categories are related to both environmental and human impact (Figs 3 and 4). The siliceous aggregate phytoliths from woody shrubs and trees are most abundant in the deposits which indicated more mesic conditions in the form of seasonally marshy floodplain deposits. They appear in Unit 1, but are most abundant in the marshy deposits of Unit 3, and the top of Unit 4. They are also prominent in the seasonal marsh deposits of Unit 5, and the lowermost part of Unit 7. These shrubs are likely responding to the increased flow and residual moisture of Tesuque Creek when it was a more perennial stream under the cooler/moister conditions of the Late Holocene [29, 56].

The distribution of succulents throughout the section shows a strong presence in the lower units but tapers off into the upper portion of the sequence. The main succulent in this region today is Yucca, specifically the *Yucca baccata* (banana yucca) which grows throughout central and western New Mexico. Although Agave plants are native to New Mexico, most are restricted to the south and southwestern portions of the state [43]. The *Yucca baccata* is particularly hearty and is more frost tolerant than other Agavoideae. It was used ethnohistorically by a number of native groups throughout the Southwest for food, medicine, utilities, and fiber [57, 58].

The fourth category of significant plant types are the dwarf palmettos (*Sabal minor*). The spheroid echinate phytoliths [59] representing this plant have almost the opposite distribution from the yucca. The palmetto phytoliths appear only in the sediments indicating moister microenvironments, and are more frequent in the upper portion of the geological section. Phytoliths from palmettos were unexpected, since there is no record in the biogeographic literature for palms or palmettos growing anywhere in this region before European contact. In order to verify this finding, we investigated the geological record and found no convincing evidence for fossil palms from any other sedimentary deposit (see S1 File). Additionally, our palmetto phytoliths are most abundant in the marshy sediment contexts and burn levels, rather than the more sandy/silt floodplain deposits, indicating their presence in the kind of microenvironment which suits their growth preference [60].

Another silica morphotype which could possibly be confused with phytoliths from palmettos are the ‘spherasters,’ silica microscleres which form in sponges [61]. These silica bodies look remarkably similar to those spheroid echinates formed in palmettos [59]. However, we feel confident that we were able to discount these sponge precipitates based on three main factors. One was that the spherasters from sponges contain a small pore either on the body of the spheroid or on the spines [62]. For the purposes of this study, Damick examined scores of our spheroid echinates under high magnification (400x) light microscopy. She then used a scanning electron microscope (SEM) and an optical profilometer to examine fifteen additional spheroid echinates from our samples and did not confirm a single pore feature in any of the forms she examined (see S1 File). Additionally, sponges produce elongated spicules at a much higher density than spherasters. The density of sponges spicules throughout all samples from TCE was extremely low, and distributed throughout the section units, whereas the spheroid echinates are very dense and concentrated within burn levels and seasonal marsh deposits. This is a strong indicator that the spheroid echinates did not derive from sponges, but rather from palmettos.

Another possibility that needed to be considered in evaluating evidence from an alluvial context is that the phytoliths were eroded from older sediments or bedrock. In our review of the geological literature, there is not compelling evidence for significant palms present in the underlying formations (one fossilized palm wood fragment) [63]. Furthermore, phytoliths, being amorphous silica, show evidence on their surfaces of chemical degradation, weathering and erosion of edges when extensively transported by wind or water, and like all minerals are subject to diagenesis [64]. However, the examples in our samples appear relatively fresh, with defined edges and distinct echinate processes. Furthermore, the fact that the spheroid echinates almost exclusively appear in the ponding levels and overlying burn layers, rather than throughout all of the sediments sampled, suggests that they were not deposited randomly by natural erosion processes but are in fact associated with those contexts. As a counter-example, several types of prismatic crystals were found consistently throughout all sediments analyzed, probably remnants of volcanic glass eroded from nearby basalts. We also measured the diameters of the TCE spheroid echinate forms from our alluvial section and compared them to those produced by Arecaceae (the palm family) in our modern reference collection, in order to determine the type of palm/palmetto with which they best compare. The only reasonable possibilities were *Sabal sp* whose range today includes Texas and Oklahoma, or *Washingtonia sp* found today in California and Arizona. The results showed that there was a highly significant statistical correlation (T = -2.11459, P = 0.035471, two-tailed) with the spheroid echinates from the *Sabal minor* palmetto, ruling out the possibility that they were from the *Washingtonia filifera* palm (See S1 File).

Given the above, we are confident that the spheroid echinate phytoliths from Tesuque Creek are almost certainly from the *Sabal minor* dwarf palmetto. These are the most cold-tolerant of the North American palms and palmettos [38]. This has significant implications for the role of humans in influencing plant distributions and native plant habitats. Since the dwarf palmetto is not native to New Mexico—and particularly not found naturally in northern New Mexico—their presence in our Late Archaic Period geological deposits demands explanation.

## Discussion

In this pilot study we investigated an alluvial drainage in northern New Mexico in order to clarify which plant resources were available to inhabitants of this region through time and how Late Archaic populations impacted the landscape and existing vegetation communities. Through phytolith analysis we were able to record some of the major vegetation changes that took place over the course of about 1,000 years during the time period in which Late Archaic populations were inhabiting this landscape. Some of these vegetation changes might have been related to the Late Holocene cooling and increase in moisture availability within the drainages of this region [29]. However, some are likely to be related to the human management of plant communities preceding the earliest local farming activities.

At the base of this alluvial section we recorded a strong presence of raphid phytoliths related to the Agavaceae subfamily. The phytolith morphology of these types are consistent with *Yucca baccata* (banana yucca), the only native Agavaceae presently growing in this region. As one of the more frost tolerant Yuccas, banana yucca is well adapted to weathering climatic extremes, and would have done well on the sandy deposits of the drier floodplain environments represented by the lower portions of the Tesuque Creek section. Scholars have known for decades that Agavaceae were important plants for Archaic peoples [65]. Their starchy fruits and stalks would have been an important source of carbohydrates in a region with relatively low-calorie content in the other plant foods, as has been the demonstrated case elsewhere [20, 66, 67].

Notably, we also found surprisingly large numbers of spheroid echinate phytoliths, which are typically abundant in palms and palmettos. Our research eliminated other possible “confusers” fossil types (see S1 File), and statistical analyses of the diameter of those spheroid echinates show that there is a high probability that they came from the cold-hardy dwarf palmetto (*Sabal minor*). We propose two possible explanations for the occurrence of *Sabal minor* phytoliths in Late Holocene sediments from Tesuque Creek. These are: a) the phytoliths represent remnant refugia groups of palmettos from earlier widespread populations that thrived during recurring short-term warming episodes within the broader regional cold period; or b) the palmettos were introduced opportunistically by Late Archaic forager communities. The latter need not have been anything close to intensive cultivation, but rather populations of foragers carrying the seeds or vegetative parts of this plant into these moist alluvial valleys and planting them there. In either scenario, whether remnants of natural refugia or anthropogenically introduced, the palmettos may have been used and maintained over time by local forager groups, a type of “low-level” cultivation in a setting where populations could visit on their yearly or seasonal rounds [1].

The greatest obstacle to understanding the presence of the palmettos in northern New Mexico is their ability to survive the paleoclimatic conditions of the Late Holocene. These conditions suggest an environment that was too cold to sustain a widespread palmetto population, even though the *Sabal* palmetto is the most cold-hardy of the palmettos. In the context of the first possible explanation for these palmettos, where they derive from natural refugia, we must look back at the evidence for their earlier presence in the region, and reconsider the variability of local climate conditions. The broad North American range of *Sabal* appears to have begun to fragment in the Eocene, continuing to gradually diminish until Pleistocene glaciation pushed it southward into its current range [68, 69]. Eventually, the Mid-Holocene Altithermal transitioned into the cooler Late Holocene, which again might have been too cold for most species of *Sabal*. However, there is clear evidence that periodic short warm episodes occurred in New Mexico during the Late Holocene Cool/Wet Period. Hall and Penner’s (2013) isotope studies of sediments from Abo Arroyo in Central New Mexico (reproduced in Hall’s 2017 Southwest Paleoclimate overview) significantly show occasional warm peaks that rival the Altithermal temperatures [29]. Armour’s 2002 study of sediments from the glacial moraines of the Sangre de Cristo Mountains also show highly variable readings for organic content, magnetic susceptibility, and δ^13^C readings, all of which could indicate temperature variability [33]. Apart from the few records we have, it can be difficult to identify high resolution patterns of temperature change within regional climatic trends. We suggest that in the case of Tesuque Creek, such periodic short-term warm episodes could easily support taxa like *Sabal* palmettos, and the appearance of palmetto phytoliths in episodic marshy deposits rather than across the entire section is consistent with the plant’s fluctuating range and abundance over the course of this time period.

In support of the second possibility for the presence of palmettos in this region, in which humans introduced them to the area opportunistically, we look to the environmental, ethnohistorical, and archaeological records of the region more broadly. The *Sabal* palmetto thrives in moist soils along streams or in wetlands and floodplains, exactly like those in which its phytoliths were found in our study section [38]. Ethnohistoric records report that the *Sabal* palmetto along such waterways was used by native peoples such as the Houma in the southern parts of North America for fiber, medicinal purposes, and importantly, the roots were roasted and eaten as a kind of bread, or transformed into a soap [70]. The small fruits were also edible. In ongoing research, A. Rosen (with T. Hart) has identified the same types of “spheroid echinate” phytoliths from *Sabal* (dwarf palmetto), in the roasting pits of the Late Paleoindian/Early Archaic Genevieve Lykes Duncan Site in the Big Bend area of West Texas. Thus, we suggest that this palmetto was moving north with forager populations very early in the Archaic, and it may well have been planted and maintained by the local pre-agricultural Late Archaic foragers even as far north as the northern Rio Grande Valley.

The maintenance required for palmetto plants is not extensive, and they are hardy in the face of many of the effects of a changing climate and human exploitation. For instance, palmetto hearts can be exploited without killing the plant, but growth would be slowed, so a yearly replacement of seed would ensure a constant supply of this resource. Palmettos are slow growing, but once started, it takes just five to seven years before the cycle can continue. Palmettos are also highly resistant to fire damage [71]. This low-maintenance and productive plant would have been an ideal early wild cultigen for mobile foragers. It would have been an important source of starchy food that could have supplemented the starches provided by the yucca found in the earlier levels of this section. As yucca might have been less adaptable to the marshy microenvironments represented by the later period deposits, palmetto may have been an appealing alternative. With the later introduction of maize agriculture in this region, the need for starchy wild plants might have decreased.

Maize starts to appear in the archaeological record of New Mexico at different points throughout the Middle to Late Archaic. The Northeastern and Southeastern Rio Grande Basin regions appear to be the latest of the populations in New Mexico to adopt full-time maize agriculture. The presence of starchy alternatives such as yucca and palmetto may be one reason why these populations did not take on maize as quickly as in other areas; Yang et al. have also suggested such a scenario to explain *Sago* palm use in pre-agricultural China [66]. Other reasons for the delayed adoption of maize agriculture could include environmental inhibitors such as too few frost-free days during this Late Holocene cool/moist episode, demographic factors such as low population densities allowing Late Archaic foragers to move freely in their home ranges, and the lack of incentives to adopt labour-intensive, high-risk agricultural activities. With the introduction of maize to this region, the need for wild sources of starch and calories would have diminished. Both yucca and palmetto not only would have fulfilled nutritional needs, but also thrive in the riverine environments already preferred by Archaic forager populations.

Another important factor to consider about the use of palmetto and yucca is that they are evergreen plants which yield important food resources all year long. Since they are available for consumption year-round, populations can rely on them when other food sources are scarce either seasonally, or in years of poor yields. Thus, the palmettos preclude the need for storage features. Ramsey and Rosen [67] describe a similar situation in the semi-arid zones of Western Asia, where foragers focused on wetlands as a source of starchy rhizomes which were available year-round, thus minimizing risk in drought prone regions. The use of these evergreen starch-rich plants may partially explain why storage features are rare in the Late Archaic Period of eastern New Mexico.

Our data supplements and extends the vast ethnohistoric literature that provides numerous examples of North American forager communities tending wild plant resources. Ethnohistoric sources show that foraging communities in California tended oak forests, silviculture was practiced by foragers in the eastern U.S. woodlands, and widespread burning to manage local ecosystems is documented in California [13–15]. Previous archaeological research in the Tesuque Creek locality supports evidence for the kinds of foraging societies which would have exploited such resources. Archaeological excavations of parts of Tesuque Creek just south of our study area have documented extensive Late Archaic features, including campsites occupied multiple times over the years as well as multiple hearth features [27, 28]. We have further identified and sampled anthropogenic burning activities (hearths and roasting pits) in the broader study area that we will be analyzing for phytolith content to see if we can trace the presence of palmettos in contexts representing direct human use. Damick has also extracted residues from grinding stones associated with Archaic material culture in these areas, which is pending analysis and publication. We can be fairly confident, then, that there was enough Late Archaic human activity in the area that they would have been familiar with these micro-environments and what they offered.

## Conclusions

In this study we have presented evidence for the presence of a previously unknown non-local plant taxon, the *Sabal* palmetto, in the floodplain of Tesuque Creek in northern New Mexico during the Late Archaic period. This has compelling implications for the management of microenvironments and plant communities, and possibly even introduction of the palmetto, by forager communities living in the area at that time. The sediments analyzed here derive from an alluvial section on the east side of Tesuque Creek dating to c. 3600–2400 cal. BP. This evidence demonstrates the value of phytolith analysis from alluvial sections for understanding human land and plant use practices over time. Wild plant cultivation by pre-agricultural foragers has long been theorized and is indirectly suggested by other data; this study adds strong evidence of this practice via the presence of an introduced wild plant that was never domesticated and does not appear to have been used by early agriculturalists in this region. Whether its introduction to the area was natural or anthropogenic, we believe the palmetto was cultivated during the Late Archaic because it was identified in an area on the landscape outside of a primary occupation site, and therefore cannot be confused with plants that were grown outside of the region and brought in after harvesting or as artifacts. Rather, the context of the palmetto phytoliths suggests that they were cultivated in this floodplain setting, in association with burning activities. This provides a new perspective on what resources and land use areas were available for Archaic peoples inhabiting the area, and how they may have experimented with managing other types of plant resources before the establishment of the triad of crops from Mesoamerica. This opens up new avenues for understanding the landscapes, land use practices, and environmental impacts of pre-agricultural communities in the northern Rio Grande and in other semi-arid environments worldwide.

## Materials and methods

Sediments were described in the field at the same time that sediment samples were taken from every unit. These samples were shipped to the Environmental Archaeology Lab at the University of Texas at Austin, where they were processed for phytoliths, counted, and recorded according to Rosen’s protocol (See S1 File). Morphotype descriptions conform as closely as possible to the International Code for Phytolith Nomenclature 2.0 [59]. Selected samples were observed under Scanning Electron Microscope and Optical Profilometer to confirm the surface microtopography of the spheroid echinate types. These methods are described in detail in the S1 File.

## Supporting information

S1 File Supporting text and tables. This file contains (1) text providing details on the processing and analysis of the phytoliths described in this research, as well as extensive details on the identification of the spheroid echinates and possible confusers for that morphotype; (2) S1 Table: Density counts (n/gram) of all phytolith morphotypes observed in the Tesuque Creek East Section samples. (3) S2 Table: Standardized measurements of Spheroid Echinates from *Washingtonia filifera*, *Sabal minor*, and the paleoecological samples from Tesuque Creek East Geo-Section, Unit 1; (4) S3 Table: All measurements of spheroid echinates from Sabal minor and Washingtonia Filifera Reference Collections and paleoecological samples from TCE.
(DOCX)

## References

[1] Smith BD. Low-level food production. Journal of Archaeological Research. 2001;9(1):1–43.

[2] Willcox G. The roots of cultivation in southwestern Asia. Science. 2013;341(6141):39–40. doi: 10.1126/science.1240496 23828931

[3] Greaves RD, Kramer KL. Hunter–gatherer use of wild plants and domesticates: archaeological implications for mixed economies before agricultural intensification. Journal of Archaeological Science. 2014;41:263–71.

[4] Piperno DR, Weiss E, Holst I, Nadel D. Processing of wild cereal grains in the Upper Palaeolithic revealed by starch grain analysis. Nature. 2004;430(7000):670. doi: 10.1038/nature02734 15295598

[5] Piperno DR, Flannery KV. The earliest archaeological maize (Zea mays L.) from highland Mexico: new accelerator mass spectrometry dates and their implications. Proceedings of the National Academy of Sciences. 2001;98(4):2101–3. doi: 10.1073/pnas.98.4.2101 11172082 PMC29388

[6] Fuller DQ, Sato Y-I, Castillo C, Qin L, Weisskopf AR, Kingwell-Banham EJ, et al. Consilience of genetics and archaeobotany in the entangled history of rice. Archaeological and Anthropological Sciences. 2010;2(2):115–31.

[7] Asouti E, Fuller DQ, Barker G, Finlayson B, Matthews R, Fazeli Nashli H, et al. A contextual approach to the emergence of agriculture in Southwest Asia: reconstructing Early Neolithic plant-food production. Current Anthropology. 2013;54(3):000–.

[8] Weiss H. The Origins of Cities in dry-farming Syria and Mesopotamia in the third Millennium BC: Four Quarters Publishing Company; 1986.

[9] Zeder MA. Domestication and early agriculture in the Mediterranean Basin: Origins, diffusion, and impact. Proceedings of the national Academy of Sciences. 2008;105(33):11597–604. 10.1073/pnas.0801317105 PMC2575338 18697943

[10] Iriarte J. New perspectives on plant domestication and the development of agriculture in the New World. Rethinking agriculture: archaeological and ethnoarchaeological perspectives. 2007:167–88.

[11] Browman DL, Fritz GJ, Watson PJ. Origins of food-producing economies in the Americas. The Human Past. 2005:306–49.

[12] Vierra BJ, Ford RI. Early maize agriculture in the northern Rio Grande valley, New Mexico. Histories of maize. 2006:497–510.

[13] Blackburn TC, Anderson K. Before the wilderness: environmental management by native Californians: Ballena Pr; 1993.

[14] Lightfoot KG, Cuthrell RQ. Anthropogenic burning and the Anthropocene in late-Holocene California. The Holocene. 2015;25(10):1581–7.

[15] Smith BD. The subsistence economies of indigenous North American societies: A handbook: Smithsonian Institution Scholarly Press; 2011.

[16] Harlan JR. Harvesting of wild-grass seed and implications for domestication. Prehistory of Agriculture: New Experimental and Ethnographic Approaches. 1999:1–5.

[17] Harris DR, Hillman GC. Foraging and farming: the evolution of plant exploitation: Routledge; 2014.

[18] Kirkpatrick DT, Ford RI. Basketmaker food plants from the Cimarron district, northeastern New Mexico. Kiva. 1977;42(3–4):257–69.

[19] Ford R. The Cultural Ecology of Jemez Cave. In: Vierra B, editor. From Mountaintop to Valley Bottom. Salt Lake City, Utah: University of Utah Press; 2013.

[20] Ryan P, Rosen A. Managing risk through diversification in plant exploitation during the seventh millennium BC. Climate and cultural change in Prehistoric Europe and the Near East. 2016:117–35.

[21] Piperno DR, Ranere AJ, Holst I, Hansell P. Starch grains reveal early root crop horticulture in the Panamanian tropical forest. Nature. 2000;407(6806):894. doi: 10.1038/35038055 11057665

[22] Vierra BJ, Ford RI. Foragers and Farmers in the Northern Rio Grande Valley, New Mexico. Kiva. 2007;73(2):117–30.

[23] Markgraf JPB V., Forester R. M., Singh G., and S. R. Sternberg San Agustin Plains, New Mexico: age and paleoenvironmental potential reassessed. Quaternary Research. 1984;22(3):336–43.

[24] Schoenwetter J. America before the European Invasions. Alice Beck Kehoe 2002. Longman/Pearson Education Ltd., London, viii+ 259 pp. $20.00 (paper), ISBN 0-582-41486-5. American Antiquity. 2005;70(4):790–1.

[25] Ford RI. Ecological consequences of early agriculture in the Southwest. Papers on the archaeology of Black Mesa, Arizona. 1984;2:127–38.

[26] Ford RI. Re-excavation of Jemez Cave, New Mexico. Awanyu. 1975;3(3):13–27.

[27] Moore JL. Land Use, Settlement, and Community in the Southern Tewa Basin, Vol. 3: The Prehistoric Sites and Site Components. Manuscript on file, Office of Archaeological Studies, Museum of New Mexico, Santa Fe; 2018. Report No.: Archaeology Notes 404.

[28] Wendorf JPMaF. Alluvial Chronology of the Tesuque Valley, New Mexico. Journal of Geology. 1958;66(2):177–94.

[29] Hall S. Paleoenvironments of the American Southwest. In: Vierra B, editor. The Archaic Southwest Foragers in an Arid Land. Salt Lake City, Utah: University of Utah Press; 2017.

[30] Baisan TSaC. Tree-ring reconstructions of fire and climate history in the Sierra Nevada and southwestern United States. In: Thomas T. Veblen WLB, Gloria Montenegro, and Swetnam Thomas W., editor. Fire and climatic change in temperate ecosystems of the western Americas (pp 158–195) Springer, New York, NY: Springer; 2006. p. 158–95.

[31] Paul Szejner WEW, Flurin Babst, Soumaya Belmecheri, Valerie Trouet, Leavitt Steven W, Ehleringer James R, Monson Russell K. Latitudinal gradients in tree ring stable carbon and oxygen isotopes reveal differential climate influences of the North American Monsoon System. Journal of Geophysical Research: Biogeosciences. 2016;121(7):1978–91.

[32] Jill Onken SJS, Palacios-Fest Manuel R., and Adams Karen R. Late Holocene hydroclimatic change at Cienega Amarilla, west-central New Mexico, USA. Quaternary Research 2017;87(2):227–45.

[33] Jake Armour PJF, John W. Geissman. 15 ky paleoclimatic and glacial record from northern New Mexico. Geology. 2002;30(8):723–6.

[34] Jiménez-Moreno PJFaRSA G. Millennial- and centennial-scale vegetation and climate changes during the late Pleistocene and Holocene from northern New Mexico. Quaternary Science Reviews. 2008;27(13–14):1442–52.

[35] Vierra BJ. From Mountain Top to Valley Bottom: Understanding Past Land Use in the Northern Rio Grande Valley, New Mexico: University of Utah Press; 2013.

[36] Vierra MMaB. The Southwest Archaic. In: Fowles BMaS, editor. The Oxford Handbook of Southwest Archaeology. Oxford, United Kingdom: Oxford University Press; 2017. p. 231–45.

[37] Swetnam TW, Betancourt JL. Mesoscale disturbance and ecological response to decadal climatic variability in the American Southwest. Journal of Climate. 1998;11(12):3128–47.

[38] Butler JLC C.J., McBride K., Arbour D., and Heck B. Modeling the distribution of the dwarf palmetto (Sabal minor; ARECACEAE) in McCurtain County, Oklahoma. The Southwestern Naturalist. 2011;56(1):66–71.

[39] Huckell BB. The archaic prehistory of the North American Southwest. Journal of World Prehistory. 1996;10(3):305–73.

[40] Irwin-Williams C. Post-Pleistocene archaeology, 7000–2000 BC. Handbook of North American Indians. 1979;9:31–42.

[41] Berry CF, Berry MS. Chronological and conceptual models of the Southwestern Archaic. Anthropology of the Desert West: Essays in Honor of Jesse D Jennings, edited by Carol J Condie and Don D Fowler. 1986:253–327.

[42] McBrinn ME. Everything old is new again: Recent approaches to research on the Archaic period in the western United States. Journal of Archaeological Research. 2010;18(3):289–329.

[43] Allred KW, Ivey RD. Flora neomexicana. 2nd ed. Raleigh, N.C.: Lulu; 2012. v. p.

[44] Castetter EF, Bell WH, Grove AR. The early utilization and the distribution of agave in the American Southwest. 1938.

[45] Suluvan AP III, Skibo JM, Van Buren M. Sherds as tools: The roles of vessel fragments in prehistoric succulent plant processing. North American Archaeologist. 1992;12(3):243–55.

[46] Tainter JA, Tainter BB. Evolving complexity and environmental risk in the prehistoric southwest. 1996.

[47] Teague LS, Teiwes H. Textiles in Southwestern Prehistory: University of New Mexico Press; 1998.

[48] Fish SK, Fish PR. Agave (Agave spp.): a crop lost and found in the US-Mexico borderlands. New Lives for Ancient and Extinct Crop. 2014:102–38.

[49] Cummings LS. Great Basin Paleoethnobotany. In: Minnis P, editor. People and plants in ancient western North America. Washington, D.C.: Smithsonian Books; 2003. p. 205.

[50] Merrill WL, Hard RJ, Mabry JB, Fritz GJ, Adams KR, Roney JR, et al. The diffusion of maize to the southwestern United States and its impact. Proceedings of the National Academy of Sciences. 2009;106(50):21019–26. doi: 10.1073/pnas.0906075106 19995985 PMC2795521

[51] Huckell LW. Ancient maize in the American Southwest. In: JE Staller RT, and Benz BF, BF, editor. Histories of Maize: Multidisciplinary Approaches to the Prehistory, Biogeography, Domestication, and Evolution of Maize: Left Coast Press; 2006. p. 97–107.

[52] McBride PaMST. From the Land o the Little Birds to the Valleys of the White Rock, Tewa, Galisteo, Rio Grande, and Santa Fe Rivers. In: Vierra B, editor. From Mountain Top to Valley Bottom: Understanding Past Land Use in the Northern Rio Grande Valley, New Mexico. Salt Lake City: University of Utah Press; 2013.

[53] Post S. Transitional archaic and emergent agricultural settlement in the lowland-upland settings of the northern Rio Grande, New Mexico. In: Vierra B, editor. From Mountaintop to Valley Bottom. Salt Lake City: University of Utah Press; 2013. p. 80–94.

[54] Vierra B. Archaic Foragers of the Northern Rio Grande Valley (No. LA-UR-07-2712). Los Alamos, NM (United States): Los Alamos National Lab (LANL); 2007.

[55] Bozarth SR. Classification of opal phytoliths formed in selected dicotyledons native to the Great Plains. Phytolith systematics: Springer; 1992. p. 193–214.

[56] French C, Periman R, Cummings LS, Hall S, Goodman‐Elgar M, Boreham J. Holocene alluvial sequences, cumulic soils and fire signatures in the middle Rio Puerco Basin at Guadalupe Ruin, New Mexico. Geoarchaeology: An International Journal. 2009;24(5):638–76.

[57] Huckell LW, Toll MS. Wild plant use in the North American Southwest. People and plants in ancient western North America Tucson: University of Arizona Press; 2004. p. 37–114.

[58] Kimmerer RW. Braiding sweetgrass: Indigenous wisdom, scientific knowledge and the teachings of plants: Milkweed Editions; 2013.

[59] Neumann K, Strömberg CA, Ball T, Albert RM, Vrydaghs L, Cummings LS. International code for phytolith nomenclature (ICPN) 2.0. Annals of Botany. 2019. doi: 10.1093/aob/mcz064 31334810 PMC6758648

[60] Zona S. The genera of Palmae (Arecaceae) in the southeastern United States. Harvard Papers in Botany. 1997;2:71–108.

[61] Yost CL. Phytolith Analysis of Early Agricultural Period Field Sediments at Las Capas, AZ AA:12:111 (ASM) Tucson: Archaeology Southwest; 2015. Report No.: Anthropological Papers No. 50.

[62] Uriz M. Mineral skeletogenesis in sponges. Canadian Journal of Zoology. 2006;84:322–56.

[63] Galusha T, and Blick, J.C. Stratigraphy of the Santa Fe Group, New Mexico. Bulletin of the American Museum of Natural History. 1971;144:1–128.

[64] Piperno DR. Phytoliths: a comprehensive guide for archaeologists and paleoecologists: Rowman Altamira; 2006.

[65] Kaplan L. Archeoethnobotany of cordova cave, New Mexico. Economic Botany. 1963;17(4):350–9.

[66] Yang X, Barton HJ, Wan Z, Li Q, Ma Z, Li M, et al. Sago-type palms were an important plant food prior to rice in southern subtropical China. PLoS One. 2013;8(5):e63148. doi: 10.1371/journal.pone.0063148 23667584 PMC3648538

[67] Ramsey MN, Rosen AM. Wedded to wetlands: Exploring Late Pleistocene plant-use in the Eastern Levant. Quaternary International. 2016;396:5–19.

[68] Zona S. A monograph of Sabal (Arecaceae: Coryphoideae). Aliso: A Journal of Systematic and Evolutionary Botany. 1990;12(4):583–666.

[69] Leopold EB, MacGinitie HD. Development and affinities of Tertiary floras in the Rocky Mountains. Floristics and Paleoflorists of Asia and Eastern North America. 1972.

[70] Castetter EF. The vegetation of New Mexico. New Mexico Quarterly. 1956;26(3):16.

[71] Haynes JaJM. Edible Palms and Their Uses. Gainesville, Florida: University of Florida Press; 2000.

