# Supplementary material for: Palm springs on the Rio Grande: Insight into Archaic forager plant use from phytoliths recovered from a Late Holocene alluvial section in northern New Mexico
Source: PLoS One. 2021 Oct 12;16(10):e0258231. doi: 10.1371/journal.pone.0258231 (PMC8509927; doi:10.1371/journal.pone.0258231)
Supplement: S1 File — This file contains (1) text providing details on the processing and analysis of the phytoliths described in this research, as well as extensive details on the identification of the spheroid echinates and possible confusers for that morphotype; (2) S1 Table: Density counts (n/gram) of all phytolith morphotypes observed in the Tesuque Creek East Section samples. (3) S2 Table: Standardized measurements of Spheroid Echinates from Washingtonia filifera, Sabal minor, and the paleoecological samples from Tesuque Creek East Geo-Section, Unit 1; (4) S3 Table: All measurements of spheroid echinates from Sabal minor and Washingtonia Filifera Reference Collections and paleoecological samples from TCE. (DOCX) [file pone.0258231.s001.docx]

**SI File: Supporting Information**

**Detailed Methods**.

*Field Geoarchaeology:*

The sediments and units were first identified and described in the field. Samples of about 100g sediment were collected at each interval to obtain a representative coverage of each distinct sediment unit. Sediment samples were double-bagged in plastic Ziploc bags, with paper labels between the bags and labels written on the outside of the outer bag. They were then sealed in plastic Tupperware containers and shipped directly to the Environmental Archaeology Lab at the University of Texas in Austin. Charcoal samples and organic-rich sediments for 14C dating were collected, sealed, and shipped in the same way.

*Unit Descriptions from Alluvial Section TCE*

The uppermost Unit 1 (0-40 cm below surface) is dated to ca. 2,700 cal BP (^14^C 2580 +/- 30 BP), and is characterized by dark organic rich clayey silts consistent with marshy deposits.

Unit 2 (40-154 cmbs) consists of sets of well-sorted near-channel pebbles, and sandy-silt laminated levee deposits.

Unit 3 (154-240 cmbs), is again characterized by more organic rich clayey-silt marshy deposits, interspersed with finely laminated sandy silt overbank sediments.

Unit 4 (240-279 cmbs) returns to more pure laminated silt and coarse sand sets with cross-bedded fine-sands at the base.

Unit 5 (279-327 cmbs) is dated to ca. 3,400 cal BP (^14^C 3380 +/- 30 BP) and consists of another layer of ponded marsh sediments with high percentages of organic matter, and silty loam texture.

Unit 6 (327-424 cmbs), is composed of fine laminated and massive silts with no evidence for ponding.

Unit 7 (424-524 cmbs) is dated to ca. 3600 cal BP ^(14^C 3440 +/- 30 BP) and consists of another combination of gleyed ponded deposits with sub-units of laminated sandy silts with occasional pebble lenses.

Unit 8 (524 cmbs with lower boundary below the modern channel) consists of massive fine-grained silts.

*Phytolith Processing Protocol*

Phytoliths were processed according to Rosen’s lab protocol. First, the sediment was sieved through a 0.25 mm sieve to remove the coarse sediment fraction. A sample of approximately 800 mg was weighed using an analytical balance and then taken for analysis. The samples were placed in clean 50 ml PVA centrifuge tubes and treated with 30 ml of 10% HCL to remove the pedogenic carbonates. Once the samples stopped reacting, indicating the removal of carbonates, the samples were washed in reverse osmosis (RO) water, before centrifuging for 5 minutes at 2000 rpm to concentrate the phytoliths and sediments at the bottom of the tube. The suspense was then poured off and this was repeated twice more to remove all the acid. To disperse the clays, 15-20 ml of sodium hexametaphosphate solution (lab grade Calgon 5% and distilled water 95%) was added to the sample. The samples were then poured into tall beakers (400 ml) and RO water added to a height of 8 cm. The samples were then mixed thoroughly with RO water and left to settle for 1 hour and 10 minutes. After settling, all of the particles larger than clay particles (~2 microns), including the phytoliths, should be resting at the bottom of the column, while the clay particles remain in suspense. The water and clay mixture was then poured off carefully, making sure to not disturb the coarse fraction with the phytoliths. RO water was then added to a height of 8 cm and the samples were again mixed thoroughly, and allowed to settle for 1 hour more, before being poured off carefully. Importantly, not all of the water gets poured off because this would result in the loss of some of the phytolith sample. The water is poured off only to a height that does not disturb the sample at the base of the water column. This settling process was repeated until the water was clear. The height of the water column (8 cm) and the time left to settle (1 hour and 10 minutes, then 1 hour subsequently) has been calculated according to Stokes’ Law, which is an equation that solves the settling velocities of spherical particles in a fluid medium. The last of the water was then pipetted out, before the samples themselves were left to dry in the drying oven at <50°C before the organic removal step. Once dry, the samples were transferred to ceramic crucibles (~10 ml) and the organic matter was removed by dry ashing the samples in a muffle furnace for 2 hours at 500°C. Once cool, the phytoliths were then extracted from the remaining fraction using density separation. A sodium polytungstate (SPT) solution (with RO water) calibrated to 2.3 specific gravity was used to separate the phytoliths from the heavier minerals. The samples were transferred from the crucibles into clean 15 ml PVA centrifuge tubes containing 3 ml of SPT and centrifuged at 800 rpm for 10 minutes. Since phytoliths have a specific gravity range between 1.5-2.3 (Prychid, et al. 2004), the phytoliths float in the heavy density liquid, while the heavier particles drop to the bottom of the centrifuge tube. The floating phytoliths at the top of the suspension were then poured into a clean 15 ml PVA centrifuge tube. RO water was then added to lower the specific gravity of the solution and the sample was then centrifuged at 2000 rpm for 5 minutes to concentrate the phytoliths at the bottom of the tube. The SPT and water dilution was then poured off and recycled, and the phytoliths washed twice more to remove any residue from the SPT. The clean phytoliths were then removed from the tubes by pipette and placed in small weighed glass beakers. Once dried and weighed, a sample of approximate 2 mg was mounted on slides in Entellan.

The phytolith slides were counted at 400x magnification using a transmitted-light microscope. A minimum of 300 single cells were counted on each slide; typically 100 multi-cells are also counted separately from single cells, but multi-cells were very rare in these samples. The absolute counts for each phytolith type was calculated using a modified method outlined by Albert, et al. (1999); Albert and Weiner (2001). The results are expressed as number per gram of sediment. The number of phytoliths on the slide was calculated using the following algorithm:

n phytoliths per slide= n counted=n slide fields counted × total n fields on slide.

This value was used to derive a comparable unit of quantification, the number of phytoliths per 1 g of sediment. This value was calculated with the following formula:

n phytoliths=g=n phytoliths per slide= total amount of sediment mounted ðmgÞ × total phytolith amount ðmgÞ= total initial sedimentðmgÞ × 1; 000.

We used this formula and an Excel spreadsheet (Microsoft) to calculate the number of each phytolith type on each slide and the number of each type per gram (see also Nadel et al 2013)The morphologies identified are described according to the ICPN protocol 1.0, with necessary adjustments made according to the ICPN protocol 2.0 (Madella 2005; Neumann et al 2019).

*SEM and Profilometer Methods*

The scanning electron microscope used was an FEI Quanta 650 ESEM and the optical profilometer was a Keyence VK-X1100 Optical Profilometer, both located in the Texas Materials Institute at the University of Texas-Austin, with the assistance of Dr. Andrei Dolocan. Wearing latex gloves, small quantities of processed archaeological samples from Tesuque Creek were mounted on carbon tape, and pressed flat with the tape backing to separate as many of the phytoliths from each other as possible and reduce crowding the images and measurements.

For the profilometer, the sample mounted on carbon tape was then directly observed at magnifications from 200x-2,000x. Optical image resolution on this instrument is low, so images are acquired via laser scanning to produce a three-dimensional model of the surface morphometrics. Fifteen archaeological examples of spheroid echinate silica bodies were observed and three that appeared optically to have potential pores on their surfaces were selected for three-dimensional image rendering. The images showed overall uneven surfaces at the micro-scale, but the profile across the area that appeared to be a pore optically did not show any significant depth when modelled.

For the SEM, the sample mounted on carbon tape was also mounted on a small metal tab which was affixed in the holder inside the main SEM body. Samples were observed with a beam spot size of 3.5-4.5 nm and a beam voltage of 30-35kV. The FEI Quanta software was used to set an observation distance of 10 mm, and to adjust the tilt, focus, and lens alignment to obtain the images provided below. Fifteen spheroid echinate bodies from the archaeological samples were observed; as none of them appeared to have pores under this high-resolution imagery, four were selected at random for imaging of the surfaces.

*Phytolith Identifications*

Most phytoliths are silt-sized micro-fossils of plant cells, usually composed of amorphous silica. They form within the epidermal tissue of grasses, sedges, and palms (Monocotyledons). They usually take on the shape of the epidermal cells in which they form, and when the organic plant matter either disintegrates or is burned, they can reside in the sediment for tens of thousands of years. Phytoliths can also form within trees, and shrubs (Dicotyledons), as well as succulents and cacti, however, these forms are less diagnostic than those formed in the Monocotyledons. Phytoliths from dicots, succulents and cacti are often composed of calcite minerals and form more crystalline shapes within the plant mesophyll tissue. These forms are called druzes and raphids depending on the crystalline shapes. All morphotypes are described using the International Code for Phytolith Nomenclature (ICPN) (59, 67).

**S1 Table***:* **Density counts (n/gram) of all phytolith morphotypes observed in the Tesuque Creek East Section samples.** All densities are derived from counts of 300 counted phytoliths, multiplied across the space of a 25x25 mm slide cover, according to the weight of total phytoliths extracted from .800 g of sediment. This is described above in further detail.

| **CM below surface** | 15 | 35 | 56 | 105 | 120 | 134 | 167 | 190 | 202 | 211 | 222 | 228 | 236 | 240 | 245 | 258 | 270 | 285 | 290 | 297 | 315 | 327 | 355 | 410 | 424 | 435 | 450 | 464 | 477 | 515 |
| --- | --- | --- | --- | --- | --- | --- | --- | --- | --- | --- | --- | --- | --- | --- | --- | --- | --- | --- | --- | --- | --- | --- | --- | --- | --- | --- | --- | --- | --- | --- |
| **Sample Number** | P1 | P2 | P3 | P4 | P5 | P6 | P7 | P8 | P9 | P10 | P11 | P12 | P13 | P14 | P15 | P16 | P17 | P18 | P19 | P20 | P21 | P22 | P23 | P24 | P25 | P26 | P27 | P28 | P29 | P30 |
| Elongate psilate | 3961 | 52975 | 1811 | 825 | 118 | 111 | 433 | 19333 | 60864 | 189009 | 15650 | 44762 | 115165 | 36721 | 175 | 0 | 1232 | 3710 | 19407 | 2648 | 67827 | 9760 | 14724 | 34501 | 858 | 1833 | 15786 | 18744 | 11503 | 2671 |
| Elongate sinuate | 0 | 0 | 0 | 0 | 0 | 0 | 0 | 2274 | 4586 | 9383 | 2197 | 2842 | 55576 | 3295 | 0 | 0 | 0 | 0 | 1401 | 0 | 17694 | 0 | 10123 | 0 | 0 | 0 | 0 | 0 | 359 | 0 |
| Long (Rods) | 704 | 0 | 0 | 0 | 0 | 0 | 0 | 0 | 0 | 0 | 0 | 0 | 0 | 5649 | 0 | 0 | 0 | 0 | 0 | 0 | 2949 | 0 | 0 | 0 | 0 | 0 | 0 | 882 | 0 | 0 |
| Elongate echinate | 528 | 18294 | 138 | 262 | 0 | 33 | 0 | 3980 | 6670 | 20107 | 1373 | 16342 | 33963 | 0 | 0 | 0 | 0 | 0 | 0 | 0 | 2949 | 945 | 17485 | 4388 | 0 | 0 | 0 | 0 | 120 | 0 |
| Elongate dendritic | 264 | 0 | 0 | 0 | 0 | 0 | 0 | 0 | 0 | 0 | 0 | 0 | 0 | 0 | 0 | 0 | 0 | 0 | 0 | 0 | 0 | 630 | 3681 | 0 | 0 | 0 | 504 | 0 | 0 | 0 |
| Acicular psilate (hairs) | 88 | 0 | 315 | 112 | 39 | 0 | 1212 | 0 | 5003 | 0 | 1647 | 0 | 3088 | 4237 | 0 | 0 | 308 | 3765 | 0 | 0 | 5898 | 315 | 9203 | 0 | 22 | 0 | 0 | 0 | 0 | 0 |
| Acicular psilate (trichomes) | 0 | 0 | 0 | 0 | 0 | 11 | 260 | 0 | 1251 | 4021 | 0 | 0 | 0 | 6120 | 44 | 0 | 0 | 2510 | 0 | 0 | 13271 | 630 | 6442 | 0 | 0 | 0 | 0 | 0 | 0 | 0 |
| Bulliform: | 704 | 3811 | 453 | 112 | 256 | 22 | 1039 | 6634 | 11673 | 16086 | 9335 | 15631 | 37050 | 12711 | 132 | 4595 | 308 | 6275 | 4202 | 0 | 45710 | 1574 | 33129 | 8713 | 1276 | 877 | 16543 | 36541 | 5512 | 1623 |
| Bulliform flabellate | 88 | 1143 | 0 | 0 | 0 | 0 | 0 | 0 | 1668 | 0 | 0 | 0 | 0 | 1412 | 0 | 0 | 0 | 0 | 12606 | 0 | 13271 | 0 | 3681 | 0 | 1232 | 0 | 345 | 0 | 2876 | 237 |
| Tabular crenate | 0 | 1143 | 0 | 0 | 0 | 0 | 0 | 0 | 5836 | 8043 | 0 | 0 | 0 | 0 | 0 | 0 | 0 | 1255 | 0 | 0 | 0 | 0 | 0 | 0 | 0 | 0 | 0 | 0 | 0 | 0 |
| Bilobates | 0 | 0 | 0 | 0 | 0 | 0 | 0 | 0 | 1251 | 0 | 0 | 0 | 5146 | 0 | 0 | 0 | 0 | 0 | 0 | 0 | 0 | 0 | 0 | 0 | 0 | 0 | 0 | 0 | 0 | 0 |
| Rondels | 792 | 2668 | 79 | 0 | 0 | 0 | 0 | 0 | 834 | 9383 | 0 | 0 | 9263 | 0 | 0 | 0 | 0 | 0 | 0 | 0 | 56031 | 0 | 11043 | 0 | 44 | 0 | 0 | 1267 | 3715 | 101 |
| Saddles | 0 | 1143 | 0 | 0 | 0 | 0 | 0 | 0 | 0 | 0 | 0 | 0 | 0 | 1412 | 0 | 0 | 0 | 0 | 0 | 0 | 8847 | 0 | 0 | 0 | 0 | 0 | 0 | 0 | 120 | 0 |
| Conical (sedge cones) | 0 | 3049 | 0 | 0 | 0 | 0 | 0 | 0 | 0 | 0 | 0 | 0 | 0 | 0 | 0 | 492 | 22 | 1673 | 1401 | 0 | 0 | 1259 | 2761 | 0 | 0 | 0 | 0 | 0 | 0 | 0 |
| Raphids | 0 | 1524 | 0 | 75 | 0 | 699 | 346 | 379 | 834 | 5362 | 0 | 6395 | 0 | 3295 | 0 | 5416 | 110 | 17571 | 13073 | 0 | 0 | 4093 | 24847 | 0 | 110 | 45043 | 4053 | 0 | 359 | 0 |
| Spheroid echinates | 968 | 17912 | 39 | 0 | 0 | 0 | 0 | 1706 | 1668 | 8043 | 549 | 0 | 0 | 0 | 0 | 0 | 22 | 418 | 2345 | 0 | 10322 | 630 | 2761 | 0 | 22 | 0 | 0 | 201 | 1678 | 0 |
| Spheroid verrucate | 176 | 2287 | 276 | 0 | 276 | 0 | 1299 | 0 | 834 | 6702 | 1922 | 0 | 3088 | 2825 | 88 | 656 | 990 | 2510 | 0 | 0 | 0 | 945 | 460 | 0 | 0 | 0 | 0 | 0 | 0 | 0 |
| Spheroid psilate | 0 | 3049 | 0 | 0 | 0 | 0 | 0 | 0 | 0 | 0 | 0 | 0 | 0 | 1883 | 0 | 0 | 0 | 5020 | 0 | 0 | 1475 | 0 | 3681 | 0 | 0 | 0 | 0 | 0 | 0 | 34 |
| Rectangular facetate | 0 | 15626 | 0 | 0 | 216 | 78 | 1559 | 3412 | 33767 | 85791 | 17572 | 50446 | 163640 | 63556 | 0 | 10504 | 748 | 18826 | 8404 | 1135 | 0 | 0 | 7822 | 0 | 0 | 478 | 0 | 0 | 479 | 0 |
| Blocky | 264 | 0 | 335 | 0 | 59 | 0 | 779 | 569 | 0 | 0 | 1647 | 5684 | 0 | 1412 | 395 | 0 | 0 | 5020 | 11672 | 0 | 0 | 0 | 0 | 6547 | 286 | 239 | 0 | 0 | 359 | 0 |
| Rectangular (platelet) | 2640 | 25916 | 1338 | 337 | 236 | 67 | 8834 | 8340 | 22928 | 44236 | 8786 | 34105 | 57634 | 13182 | 395 | 14114 | 2046 | 22592 | 26612 | 13430 | 5898 | 12909 | 19325 | 358021 | 374 | 1435 | 36421 | 0 | 4194 | 3889 |
| Tabular foliate (compound platelet) | 1584 | 1906 | 354 | 262 | 610 | 11 | 7621 | 2843 | 23345 | 48258 | 13179 | 40499 | 18525 | 3766 | 1623 | 5580 | 616 | 5857 | 6536 | 3026 | 0 | 7242 | 0 | 44870 | 0 | 956 | 0 | 0 | 0 | 1116 |
| Acicular elongate (pinus type) | 0 | 0 | 0 | 0 | 0 | 0 |  |  |  |  | 0 | 0 | 0 | 0 | 0 | 0 | 0 | 0 | 0 | 0 | 2949 | 0 | 0 | 0 | 0 | 0 | 0 | 0 | 0 | 0 |
| silica aggregate | 616 | 4192 | 630 | 900 | 0 | 239 | 953 | 1327 | 1251 | 8043 | 11532 | 25579 | 6175 | 16948 | 88 | 5744 | 1518 | 8786 | 8871 | 1135 | 0 | 3463 | 6902 | 0 | 2310 | 438 | 721 | 0 | 5512 | 101 |
| Polyhedral | 0 | 0 | 0 | 0 | 0 | 0 | 2085 | 3335 | 9383 | 824 | 6395 | 0 | 4237 | 0 | 175 | 0 | 44 | 2801 | 0 | 0 | 0 | 0 | 0 | 0 | 0 | 0 | 0 | 0 | 0 | 0 |
| **TOTAL** | **13378** | **156639** | **5767** | **2887** | **1811** | **1270** | **26421** | **54131** | **193644** | **463292** | **91784** | **242285** | **512549** | **178426** | **3115** | **47103** | **7964** | **108592** | **116529** | **21375** | **255089** | **44394** | **178070** | **457040** | **6534** | **51300** | **74373** | **57635** | **36787** | **9772** |

*S5. Spheroid echinate silica bodies in sediment: an assessment of alternative possibilities*

In making the claim to have identified palmettos in Archaic sediments from northern New Mexico, we wanted to be as sure as possible of our findings. We sought to uncover any other references to similar silicate micro-fossils found in sediments from the region, and to investigate any other possibilities for their identification. In doing so, we came up with the following possible alternatives, in order of likelihood: spherasters from freshwater sponges (most likely *Spongilla sp.*), reported spheroid echinates identified as *Euphorbiaceae*, *Equisetum,* druse idioblasts, or taxa further afield such as *Bromeliaceae* or *Zingiberales (61, 68, 69)*.

*Equisetum* produces semi-spherical echinate phytoliths; however, the examples from our samples are fully globular, and none of the other diagnostic phytoliths from *Equisetum* (such as the characteristic stomata) are present. We could find no evidence for spheroid echinate morphotypes in *Euphorbiaceae* reference collections, nor any published images. Druse idioblasts, in addition to having a different morphology, are calcium oxalates, which are birefringent under cross-polarization. The micro-fossils presented here are amorphous under cross-polarization, and therefore are silicates. The most similar phytolith morphotypes are found in *Bromeliaceae* and various *Zingeberales* taxa*,* but the former are endemic to South America and West Africa only, while the latter are endemic to subtropical areas of Central and South America, Asia, and Africa. It is far more likely that palmettos arrived in northern New Mexico via Texas, Mexico, or the borderlands, along similar pathways to other Mesoamerican cultigens, rather than *Bromeliaceae* or *Zingiberales* traveling from significantly farther afield.

The most likely alternative identification for the phytoliths presented here is not another plant, but rather that they are microscleres known as “spherasters,” produced by freshwater sponges, in this case probably *Spongilla sp.*  While marine sponges produce much larger spherasters with more distinct points, the only positive distinguishing feature between palm phytoliths and freshwater sponge spherasters seems to be a tiny micro-pore that appears on the surfaces of sponge spherasters, whereas the palmetto phytoliths have entirely closed and generally smooth surfaces. However, surface deformations can happen on phytoliths as a result of taxonomic or laboratory processes. Furthermore, examples of spheroid echinates observed in reference collections of *Sabal minor* and *Washingtonia filifera* also sometimes appeared to have pores on the surfaces, identical to those illustrated as sponge spherasters elsewhere, under regular light microscopy. Given this, and since the phytoliths average c.5-15 microns wide, a micro-pore on the surface is far too small and unclear to be positively identified under normal optical microscopy - it looks too much like a fragment of debris in the mounting agent, or a degraded spot on the phytolith surface.

We therefore observed the paleoenvironmental samples from Tesuque Creek, as well as reference material from *Sabal minor,* under scanning electron microscope (SEM) and optical profilometer (described in detail in the Methods Section above). The SEM was used to obtain high-resolution images of the surfaces of multiple specimens to reduce the likelihood that observations were only obtained from particles that happened to be situated with the pore facing away from the beam or lens. 15 total spheroid echinate particles from archaeological contexts were observed, and no pores were seen (see images). Archaeological material from these same samples was then observed under the optical profilometer to measure the surface from multiple angles and obtain three-dimensional profile renderings of the micromorphology of any surfaces that looked like they might have pores on them. Any circular or sub-circular features on the surface of sheroid echinates observed were shown to have only very shallow depth, consistent with the micro-topography of the rest of the phytolith surface and not indicative of a distinctive pore.

In addition to these analyses, we must consider the fact that sponge spherasters typically occur in contexts where other indices of sponges (i.e., the megascleres known as “spicules” or gemmoscleres) are also present. Our samples contain almost no spicules or other sponge microfossil forms, distributed rarely through the entire Tesuque Creek section, while the spheroid echinates are very dense and correlate with marshy and burn levels specifically.. It should also be noted that *Spongilla* spp. in general have been shown to prefer deeper habitats that are permanently saturated and to be negatively correlated with high sediment loads in their habitats; while they can tolerate shallow water and currents, they do better in deeper water with more space, less turbidity, and less discontinuous/seasonal water coverage (70).

Additionally, we researched the evidence for fresh-water sponges in the waterways of New Mexico and Texas, and found that the two potential species of sponge (*Spongilla cenota* or *Spongilla lacustris*) are found in lakes or within perennial stream channels, and thrive by adhering to a hard substrate such as rocks or submerged tree trunks (71). Since the sediments in these units are fine-grained with no large rocks or pebbles, their depositional environments would not have provided the ideal living conditions for sponges. The other possibility is that sponges were growing within the stream channel itself at a time contemporary with the floodplain deposits in our section, and the spherasters were deposited with the overbank floodwaters. We can likewise rule this out as a factor, since the spheroid echinate of our deposits are not associated with all of our floodplain facies. They appear primarily within our more marshy seasonal floodplain deposits (see Fig. 4) which significantly represent a habitat favored by the dwarf palmetto (60, 72). Finally, spherasters are a form of microsclere produced in sponges at a lower density than larger, more robust megasclere spicules or gemmoscleres, which are other silica microfossils produced by sponges (71). Therefore, one would expect to find a greater number of spicules than spherasters in contexts where sponges decayed.

Another possibility that needed to be considered in evaluating evidence from an alluvial context is that the phytoliths were eroded from older sediments or bedrock. In our review of the geological literature, there is not compelling evidence for significant palms present in the underlying formations (one fossilized palm wood fragment) (73). Furthermore, phytoliths, being amorphous silica, show evidence on their surfaces of chemical degradation, weathering and erosion of edges when extensively transported by wind or water, and like all minerals are subject to diagenesis (74). However, the examples in our samples appear relatively fresh, with defined edges and distinct echinate processes. Furthermore, the fact that the spheroid echinates almost exclusively appear in the ponding levels and overlying burn layers, rather than throughout all of the sediments sampled, suggests that they were not deposited randomly by natural erosion processes but are in fact associated with those contexts. As a counter-example, several types of prismatic crystals were found consistently throughout all sediments analyzed, probably remnants of volcanic glass eroded from nearby basalts.

We then measured the diameters of 42 spheroid echinates from the sediment samples of the TCE sediment section, and ran a Student’s T-test comparing the paleoecological samples with the references for total (bark plus leaf) *Sabal minor* and total *Washingtonia filifera*. The results showed highly significant differences between the paleoecological samples and *Washingtonia* *filifera* (T=-2.11459, P=0.035471, two-tailed), and a close similarity to the spheroid echinates from *Sabal minor* (T=0.63273, P=0.527504, two-tailed). We conclude from this that the paleoecological samples are from *Sabal minor* (see Table S2).

**S2 Table: Standardized measurements of Spheroid Echinates from *Washingtonia filifera*, *Sabal minor*, and the paleoecological samples from Tesuque Creek East Geo-Section, Unit 1.**

**S3 Table: All measurements of spheroid echinates from Sabal minor and Washingtonia Filifera Reference Collections and paleoecological samples from TCE.**

|  | **Sabal Bark** | **Sabal Leaf** | **Washingtonia Bark** | **Washingtonia Leaf** | **All Sabal** | **All Washingtonia** | **Paleoecological samples from TCE** |
| --- | --- | --- | --- | --- | --- | --- | --- |
| **Item** | Diameter |  |  |  |  |  |  |
| **1** | 6.61 | 10.06 | 12.71 | 8.95 | 6.61 | 12.71 | 15.32 |
| **2** | 4.14 | 7.43 | 8.84 | 8.01 | 4.14 | 8.84 | 9.5 |
| **3** | 7.29 | 10.46 | 10.58 | 10.01 | 7.29 | 10.58 | 11.9 |
| **4** | 5.34 | 10.58 | 15.05 | 10.69 | 5.34 | 15.05 | 8.3 |
| **5** | 6.56 | 8.98 | 12.5 | 7.77 | 6.56 | 12.5 | 9.98 |
| **6** | 9.31 | 6.33 | 13.3 | 6.8 | 9.31 | 13.3 | 7.75 |
| **7** | 7.07 | 11.54 | 12.44 | 8.83 | 7.07 | 12.44 | 8.46 |
| **8** | 8.38 | 10.84 | 12.38 | 7.54 | 8.38 | 12.38 | 7.93 |
| **9** | 7.19 | 12.38 | 14.35 | 8.91 | 7.19 | 14.35 | 8.86 |
| **10** | 7.76 | 12.7 | 12.7 | 6.27 | 7.76 | 12.7 | 12.15 |
| **11** | 8.02 | 9.8 | 11.76 | 8.43 | 8.02 | 11.76 | 8.14 |
| **12** | 6.31 | 11.44 | 12.54 | 8.02 | 6.31 | 12.54 | 9.96 |
| **13** | 6.61 | 15.08 | 16.14 | 6.56 | 6.61 | 16.14 | 12.23 |
| **14** | 6.24 | 15.31 | 15.97 | 7.48 | 6.24 | 15.97 | 7.39 |
| **15** | 6.32 | 11.98 | 11.9 | 6.71 | 6.32 | 11.9 | 9.11 |
| **16** | 6.87 | 13.37 | 12.5 | 10.17 | 6.87 | 12.5 | 11.12 |
| **17** | 7.53 | 8.99 | 12.05 | 8.79 | 7.53 | 12.05 | 15.02 |
| **18** | 7.32 | 9.91 | 10.58 | 7.04 | 7.32 | 10.58 | 8.47 |
| **19** | 7.56 | 7.17 | 15.02 | 7.24 | 7.56 | 15.02 | 8.19 |
| **20** | 7.77 | 10.84 | 11.9 | 9.72 | 7.77 | 11.9 | 9.76 |
| **21** | 7.39 | 8.95 | 12.37 | 8.51 | 7.39 | 12.37 | 12.33 |
| **22** | 9.91 | 11.68 | 10.78 | 6.91 | 9.91 | 10.78 | 8.94 |
| **23** | 5.1 | 10.95 | 10.75 | 8.03 | 5.1 | 10.75 | 5.17 |
| **24** | 6.72 | 11.07 | 13.65 | 8.5 | 6.72 | 13.65 | 8.71 |
| **25** | 6.41 | 8.34 | 10.58 | 8.27 | 6.41 | 10.58 | 8.94 |
| **26** | 6.7 | 7.93 | 11.37 | 9.24 | 6.7 | 11.37 | 6.85 |
| **27** | 8.05 | 10.01 | 16.91 | 9.98 | 8.05 | 16.91 | 7.71 |
| **28** | 6.31 | 7.3 | 14.62 | 6.95 | 6.31 | 14.62 | 7.12 |
| **29** | 8.05 | 11 | 10.44 | 9.04 | 8.05 | 10.44 | 10.99 |
| **30** | 8.72 | 10.46 | 12.71 | 8.68 | 8.72 | 12.71 | 6.34 |
| **31** | 6.48 | 11.88 | 11.35 | 8.8 | 6.48 | 11.35 | 8.21 |
| **32** | 7.4 | 8.31 | 7.76 | 8.53 | 7.4 | 7.76 | 8.75 |
| **33** | 6.87 | 10.95 | 11.21 | 5.77 | 6.87 | 11.21 | 6.33 |
| **34** | 6 | 9.43 | 13.05 | 9.91 | 6 | 13.05 | 12.76 |
| **35** | 6.43 | 7.3 | 14.11 | 8.68 | 6.43 | 14.11 | 6.16 |
| **36** | 6.87 | 10.14 | 14.23 | 8.91 | 6.87 | 14.23 | 9.94 |
| **37** | 7.39 | 11.74 | 15.53 | 9.01 | 7.39 | 15.53 | 8.66 |
| **38** | 7.56 | 8.7 | 11.19 | 8.07 | 7.56 | 11.19 | 8.46 |
| **39** | 6.8 | 13.81 | 14.86 | 8.04 | 6.8 | 14.86 | 5.88 |
| **40** | 7.56 | 10.33 | 7.88 | 6.67 | 7.56 | 7.88 | 6.19 |
| **41** | 5.35 | 12.61 | 9.25 | 8.95 | 5.35 | 9.25 | 7.29 |
| **42** | 5.5 | 10.83 | 17.14 | 7.35 | 5.5 | 17.14 | 6.5 |
| **43** | 6.96 | 13.84 | 12.65 | 7.04 | 6.96 | 12.65 |  |
| **44** | 7.22 | 11.4 | 8.22 | 7.54 | 7.22 | 8.22 |  |
| **45** | 8.36 | 11.79 | 13.04 | 8.95 | 8.36 | 13.04 |  |
| **46** | 6.35 | 10.87 | 11.54 | 9.98 | 6.35 | 11.54 |  |
| **47** | 7.29 | 9.92 | 8.9 | 9.24 | 7.29 | 8.9 |  |
| **48** | 7.43 | 11.54 | 11.54 | 8.53 | 7.43 | 11.54 |  |
| **49** | 7.54 | 8.1 | 6.84 | 10.64 | 7.54 | 6.84 |  |
| **50** | 6.61 | 7.8 | 5.3 | 8.91 | 6.61 | 5.3 |  |
| **51** | 10.75 | 12.4 | 13.48 | 7.72 | 10.75 | 13.48 |  |
| **52** | 5.12 | 7.59 | 14.49 | 8.55 | 5.12 | 14.49 |  |
| **53** | 7.3 | 8.62 | 13.58 | 6.8 | 7.3 | 13.58 |  |
| **54** | 7.99 | 11.52 | 13.78 | 10.78 | 7.99 | 13.78 |  |
| **55** | 6.13 | 11.67 | 6.8 | 8.5 | 6.13 | 6.8 |  |
| **56** | 8.34 | 11.75 | 7.28 | 8.02 | 8.34 | 7.28 |  |
| **57** | 8.25 | 10.54 | 19.36 | 9.12 | 8.25 | 19.36 |  |
| **58** | 4.91 | 11.54 | 11.96 | 7.77 | 4.91 | 11.96 |  |
| **59** | 7.82 | 11.84 | 11.45 | 8.98 | 7.82 | 11.45 |  |
| **60** | 6.84 | 10.01 | 12.9 | 9.48 | 6.84 | 12.9 |  |
| **61** | 8.48 | 9.71 | 6.31 | 7.77 | 8.48 | 6.31 |  |
| **62** | 7.84 | 11.64 | 9.62 | 8.75 | 7.84 | 9.62 |  |
| **63** | 8.8 | 9.31 | 18.48 | 9.47 | 8.8 | 18.48 |  |
| **64** | 7.17 | 10.93 | 10.67 | 7.72 | 7.17 | 10.67 |  |
| **65** | 8.77 | 13.49 | 6.22 | 10.3 | 8.77 | 6.22 |  |
| **66** | 5.25 | 8.37 | 12.22 | 8.89 | 5.25 | 12.22 |  |
| **67** | 8.58 | 10.25 | 12.75 | 8.75 | 8.58 | 12.75 |  |
| **68** | 6.88 | 14.59 | 11.23 | 6.84 | 6.88 | 11.23 |  |
| **69** | 5.79 | 12.62 | 14.96 | 7.29 | 5.79 | 14.96 |  |
| **70** | 6.6 | 10.91 | 11.33 | 6.43 | 6.6 | 11.33 |  |
| **71** | 7.09 | 12.14 | 10.22 | 7.76 | 7.09 | 10.22 |  |
| **72** | 3.79 | 11.91 | 15.61 | 6.91 | 3.79 | 15.61 |  |
| **73** | 7.82 | 8.5 | 13.36 | 9.43 | 7.82 | 13.36 |  |
| **74** | 7.43 | 12.37 | 9.43 | 7.5 | 7.43 | 9.43 |  |
| **75** | 5.15 | 10.73 | 7.08 | 8.37 | 5.15 | 7.08 |  |
| **76** | 4.32 | 13.5 | 12.02 | 7.08 | 4.32 | 12.02 |  |
| **77** | 4.56 | 12.71 | 11.18 | 6.32 | 4.56 | 11.18 |  |
| **78** | 6.35 | 11.04 | 12.39 | 7.99 | 6.35 | 12.39 |  |
| **79** | 6.99 | 14.33 | 13.49 | 7.72 | 6.99 | 13.49 |  |
| **80** | 6.54 | 10.27 | 12.67 | 6.3 | 6.54 | 12.67 |  |
| **81** | 6.84 | 10.99 | 8.6 | 8.47 | 6.84 | 8.6 |  |
| **82** | 7.38 | 9.15 | 13.68 | 7.83 | 7.38 | 13.68 |  |
| **83** | 5.87 | 10.25 | 7.77 | 6.8 | 5.87 | 7.77 |  |
| **84** | 4.92 | 5.01 | 8.07 | 8.75 | 4.92 | 8.07 |  |
| **85** | 5 | 9.01 | 10.24 | 6.76 | 5 | 10.24 |  |
| **86** | 7.24 | 5.24 | 11.66 | 7.68 | 7.24 | 11.66 |  |
| **87** | 4.91 | 10.73 | 10.48 | 9.02 | 4.91 | 10.48 |  |
| **88** | 4.34 | 11.92 | 15.54 | 7.17 | 4.34 | 15.54 |  |
| **89** | 7.11 | 8.75 | 9.47 | 9.74 | 7.11 | 9.47 |  |
| **90** | 6.32 | 7.04 | 12.27 | 7.56 | 6.32 | 12.27 |  |
| **91** | 5.63 | 12.75 | 10.84 | 8.53 | 5.63 | 10.84 |  |
| **92** | 5.83 | 9.71 | 15.78 | 5.63 | 5.83 | 15.78 |  |
| **93** | 6.31 | 11.54 | 9.24 | 10.33 | 6.31 | 9.24 |  |
| **94** | 5.91 | 13.63 | 8.67 | 8.91 | 5.91 | 8.67 |  |
| **95** | 7.01 | 16.57 | 7.8 | 8.77 | 7.01 | 7.8 |  |
| **96** | 9.01 | 11.96 | 8.43 | 6.73 | 9.01 | 8.43 |  |
| **97** | 8.01 | 11.94 | 11.26 | 6.84 | 8.01 | 11.26 |  |
| **98** | 3.89 | 8.07 | 12.05 | 6.63 | 3.89 | 12.05 |  |
| **99** | 5.19 | 12.44 | 10.45 | 6.08 | 5.19 | 10.45 |  |
| **100** | 7.3 | 5.77 | 10.44 | 7.06 | 7.3 | 10.44 |  |
| **101** | 6.67 | 10.43 | 8.77 | 7.53 | 6.67 | 8.77 |  |
| **102** | 6.14 | 9 | 15.62 | 7.84 | 6.14 | 15.62 |  |
|  |  |  | 14.53 | 8.91 | 10.06 | 14.53 |  |
|  |  |  |  |  | 7.43 | 8.95 |  |
|  |  |  |  |  | 10.46 | 8.01 |  |
|  |  |  |  |  | 10.58 | 10.01 |  |
|  |  |  |  |  | 8.98 | 10.69 |  |
|  |  |  |  |  | 6.33 | 7.77 |  |
|  |  |  |  |  | 11.54 | 6.8 |  |
|  |  |  |  |  | 10.84 | 8.83 |  |
|  |  |  |  |  | 12.38 | 7.54 |  |
|  |  |  |  |  | 12.7 | 8.91 |  |
|  |  |  |  |  | 9.8 | 6.27 |  |
|  |  |  |  |  | 11.44 | 8.43 |  |
|  |  |  |  |  | 15.08 | 8.02 |  |
|  |  |  |  |  | 15.31 | 6.56 |  |
|  |  |  |  |  | 11.98 | 7.48 |  |
|  |  |  |  |  | 13.37 | 6.71 |  |
|  |  |  |  |  | 8.99 | 10.17 |  |
|  |  |  |  |  | 9.91 | 8.79 |  |
|  |  |  |  |  | 7.17 | 7.04 |  |
|  |  |  |  |  | 10.84 | 7.24 |  |
|  |  |  |  |  | 8.95 | 9.72 |  |
|  |  |  |  |  | 11.68 | 8.51 |  |
|  |  |  |  |  | 10.95 | 6.91 |  |
|  |  |  |  |  | 11.07 | 8.03 |  |
|  |  |  |  |  | 8.34 | 8.5 |  |
|  |  |  |  |  | 7.93 | 8.27 |  |
|  |  |  |  |  | 10.01 | 9.24 |  |
|  |  |  |  |  | 7.3 | 9.98 |  |
|  |  |  |  |  | 11 | 6.95 |  |
|  |  |  |  |  | 10.46 | 9.04 |  |
|  |  |  |  |  | 11.88 | 8.68 |  |
|  |  |  |  |  | 8.31 | 8.8 |  |
|  |  |  |  |  | 10.95 | 8.53 |  |
|  |  |  |  |  | 9.43 | 5.77 |  |
|  |  |  |  |  | 7.3 | 9.91 |  |
|  |  |  |  |  | 10.14 | 8.68 |  |
|  |  |  |  |  | 11.74 | 8.91 |  |
|  |  |  |  |  | 8.7 | 9.01 |  |
|  |  |  |  |  | 13.81 | 8.07 |  |
|  |  |  |  |  | 10.33 | 8.04 |  |
|  |  |  |  |  | 12.61 | 6.67 |  |
|  |  |  |  |  | 10.83 | 8.95 |  |
|  |  |  |  |  | 13.84 | 7.35 |  |
|  |  |  |  |  | 11.4 | 7.04 |  |
|  |  |  |  |  | 11.79 | 7.54 |  |
|  |  |  |  |  | 10.87 | 8.95 |  |
|  |  |  |  |  | 9.92 | 9.98 |  |
|  |  |  |  |  | 11.54 | 9.24 |  |
|  |  |  |  |  | 8.1 | 8.53 |  |
|  |  |  |  |  | 7.8 | 10.64 |  |
|  |  |  |  |  | 12.4 | 8.91 |  |
|  |  |  |  |  | 7.59 | 7.72 |  |
|  |  |  |  |  | 8.62 | 8.55 |  |
|  |  |  |  |  | 11.52 | 6.8 |  |
|  |  |  |  |  | 11.67 | 10.78 |  |
|  |  |  |  |  | 11.75 | 8.5 |  |
|  |  |  |  |  | 10.54 | 8.02 |  |
|  |  |  |  |  | 11.54 | 9.12 |  |
|  |  |  |  |  | 11.84 | 7.77 |  |
|  |  |  |  |  | 10.01 | 8.98 |  |
|  |  |  |  |  | 9.71 | 9.48 |  |
|  |  |  |  |  | 11.64 | 7.77 |  |
|  |  |  |  |  | 9.31 | 8.75 |  |
|  |  |  |  |  | 10.93 | 9.47 |  |
|  |  |  |  |  | 13.49 | 7.72 |  |
|  |  |  |  |  | 8.37 | 10.3 |  |
|  |  |  |  |  | 10.25 | 8.89 |  |
|  |  |  |  |  | 14.59 | 8.75 |  |
|  |  |  |  |  | 12.62 | 6.84 |  |
|  |  |  |  |  | 10.91 | 7.29 |  |
|  |  |  |  |  | 12.14 | 6.43 |  |
|  |  |  |  |  | 11.91 | 7.76 |  |
|  |  |  |  |  | 8.5 | 6.91 |  |
|  |  |  |  |  | 12.37 | 9.43 |  |
|  |  |  |  |  | 10.73 | 7.5 |  |
|  |  |  |  |  | 13.5 | 8.37 |  |
|  |  |  |  |  | 12.71 | 7.08 |  |
|  |  |  |  |  | 11.04 | 6.32 |  |
|  |  |  |  |  | 14.33 | 7.99 |  |
|  |  |  |  |  | 10.27 | 7.72 |  |
|  |  |  |  |  | 10.99 | 6.3 |  |
|  |  |  |  |  | 9.15 | 8.47 |  |
|  |  |  |  |  | 10.25 | 7.83 |  |
|  |  |  |  |  | 5.01 | 6.8 |  |
|  |  |  |  |  | 9.01 | 8.75 |  |
|  |  |  |  |  | 5.24 | 6.76 |  |
|  |  |  |  |  | 10.73 | 7.68 |  |
|  |  |  |  |  | 11.92 | 9.02 |  |
|  |  |  |  |  | 8.75 | 7.17 |  |
|  |  |  |  |  | 7.04 | 9.74 |  |
|  |  |  |  |  | 12.75 | 7.56 |  |
|  |  |  |  |  | 9.71 | 8.53 |  |
|  |  |  |  |  | 11.54 | 5.63 |  |
|  |  |  |  |  | 13.63 | 10.33 |  |
|  |  |  |  |  | 16.57 | 8.91 |  |
|  |  |  |  |  | 11.96 | 8.77 |  |
|  |  |  |  |  | 11.94 | 6.73 |  |
|  |  |  |  |  | 8.07 | 6.84 |  |
|  |  |  |  |  | 12.44 | 6.63 |  |
|  |  |  |  |  | 5.77 | 6.08 |  |
|  |  |  |  |  | 10.43 | 7.06 |  |
|  |  |  |  |  | 9 | 7.53 |  |
|  |  |  |  |  |  | 7.84 |  |
|  |  |  |  |  |  | 8.91 |  |
